# A Fatal Case of Systemic Calciphylaxis in the Gastrointestinal Tract: A Case Report and Literature Review

**DOI:** 10.7759/cureus.36641

**Published:** 2023-03-24

**Authors:** Justine Chinnappan, Jesus Aguirre, Huda Marcus, Qazi Azher, Ghassan Bachuwa

**Affiliations:** 1 Internal Medicine, Hurley Medical Center - Michigan State University, Flint, USA; 2 Pathology, Hurley Medical Center - Michigan State University, Flint, USA

**Keywords:** end-stage renal disease (esrd), tertiary hyperparathyroidism, bowel ischemia, penile calciphylaxis, gastrointestinal calciphylaxis

## Abstract

Calciphylaxis is an infrequent yet lethal disease often associated with end-stage kidney disease (ESKD). The most common sites include proximal and distal extremities and the trunk, with few reported in the penis and very few as gastrointestinal (GI) disease. We report a case of systemic calciphylaxis in a middle-aged male, presenting with a colostomy leak and parastomal abscess. Workup revealed severe calcification of the intestinal arteries and ischemic colon necrosis. The patient underwent colectomy, antibiotic therapy, regular hemodialysis (HD), and sodium thiosulphate infusion with clinical stability. Histopathology of the colon revealed ischemic necrosis and pericolonic vessel calcification suggestive of calciphylaxis. It is an important differential to be considered in patients with risk factors presenting with symptoms of gastrointestinal hemorrhage and necrosis with perforation.

## Introduction

Calciphylaxis, also known as calcific uremic arteriolopathy (CUA), is an infrequent disorder often associated with end-stage kidney disease (ESKD). It clinically presents as skin ischemia and necrosis [1]. The mortality rate is very high, with an estimated six-month mortality of around 57% [2]. Although it is primarily reported to involve the skin, visceral manifestations have always been reported; it is just that it is rare compared to the extremities, and very few case reports have reported gastrointestinal (GI) manifestations [3-11]. We report a patient with ESKD, who is noncompliant with treatment, with severe systemic calciphylaxis presenting with a parastomal abscess and was found to have ischemic necrosis of the colon.

## Case presentation

**Figure 1:**
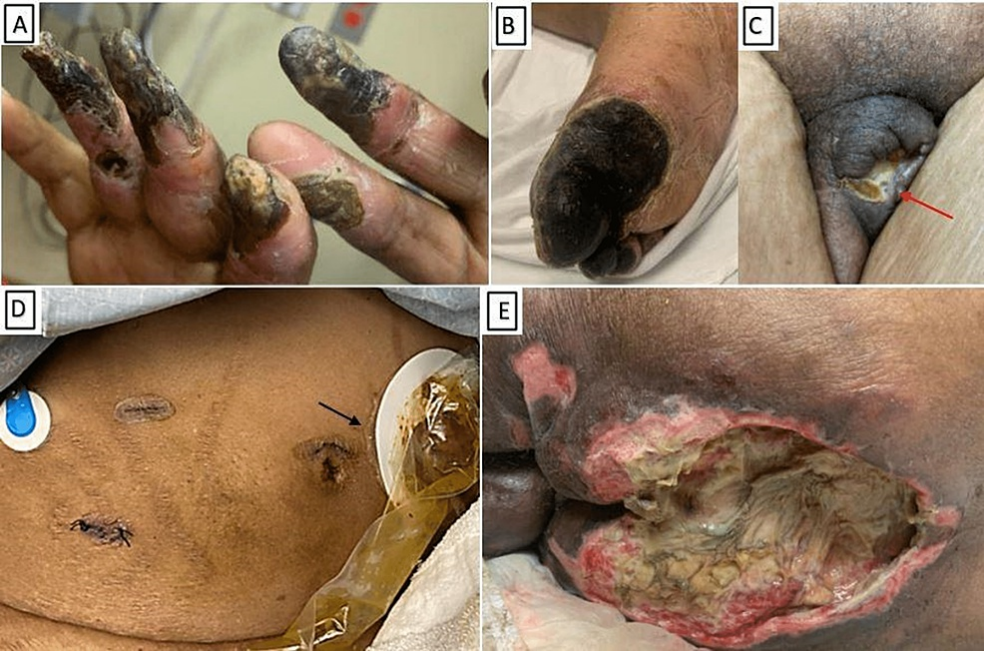
Physical examination findings A and B: Dry gangrene of the digits and right foot with black eschar. C: Post-penectomy wound (red arrow). D: Abdomen showing laparoscopic scar and leak (black arrow) around the colostomy bag. E: Stage IV infected sacral decubitus ulcer.

A 48-year-old male with essential hypertension, end-stage kidney disease on hemodialysis (HD) for nine months secondary to type 2 diabetes mellitus (DM), ESKD complicated with secondary hyperparathyroidism, penile calciphylaxis requiring partial penile amputation, diverting colostomy for sacral decubitus ulcer, and poor treatment adherence presented with worsening sacral ulcer and colostomy lip leak. He was hemodynamically stable on presentation. A thorough examination revealed a pale colostomy with stool leak around the colostomy bag, abdominal tenderness with induration around the colostomy without erythema or fluctuance, and a foul-smelling sacral ulcer that was deep, especially in the left lateral hip (Figure 1). Black discoloration of multiple digits in both hands and feet was also noted. Removal of the colostomy bag revealed a pale gray ostomy with yellow-colored foul-smelling pus draining around the stroma. Laboratory workup was significant for calcium corrected for albumin of 8.5 mg/dL (normal range: 8.6-10.3 mg/dL), serum parathyroid hormone of 112 pg/mL (normal range: 10-55 pg/mL), phosphorus of 2.4 mg/dL (normal range: 3.4-4.5 mg/dL), no leukocytosis, and elevated C-reactive protein of 76 mg/L (normal range: <0.3 mg/dL).

**Figure 2:**
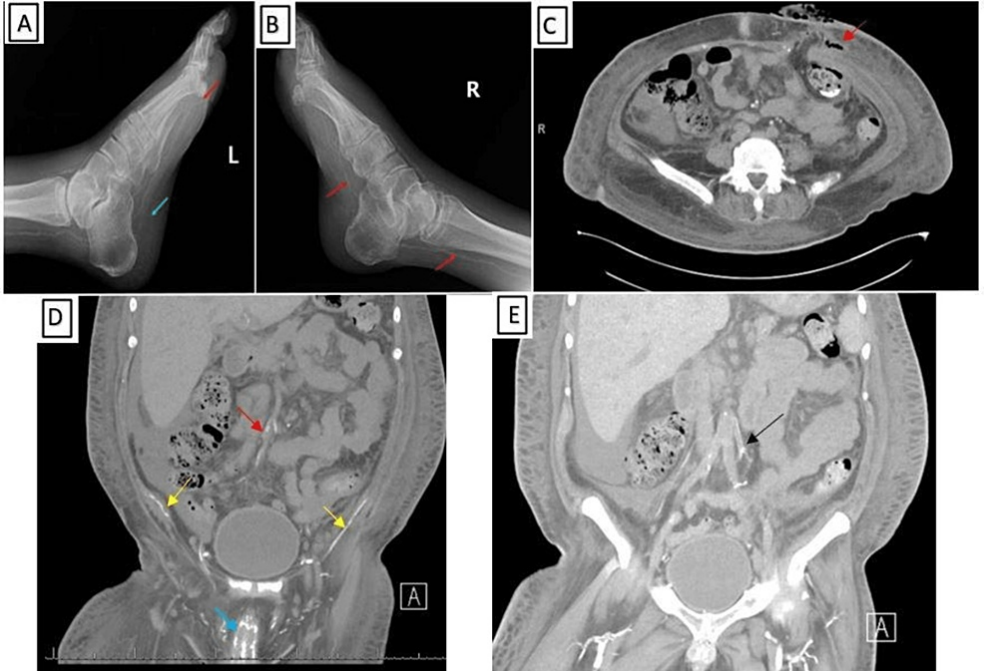
Radiological findings A and B: Left and right foot X-rays showing calcification of blood vessels (red arrows) and capillaries with reticular pattern within the soft tissue (blue arrow). C: CT scan of the abdomen and pelvis (axial) showing gas in the soft tissue (red arrow) at the site of colostomy lip. D and E: CT of the abdomen and pelvis (coronal) showing abdominal vessel calcification (red arrow), pelvis vessel calcification (yellow arrow), penile vessel calcification (blue arrow), and lead pipe calcification of the inferior mesenteric artery (black arrow). CT: computed tomography

X-ray of the bilateral foot revealed significant arterial calcification. Computed tomography (CT) of the abdomen and pelvis was remarkable for gas collection within soft tissue around the left side of the colostomy concerning for peristomal abscess and extensive calcification of abdominal blood vessels (Figure 2). Arterial Doppler of the bilateral upper and lower extremity was significant for extensive calcification.

**Figure 3:**
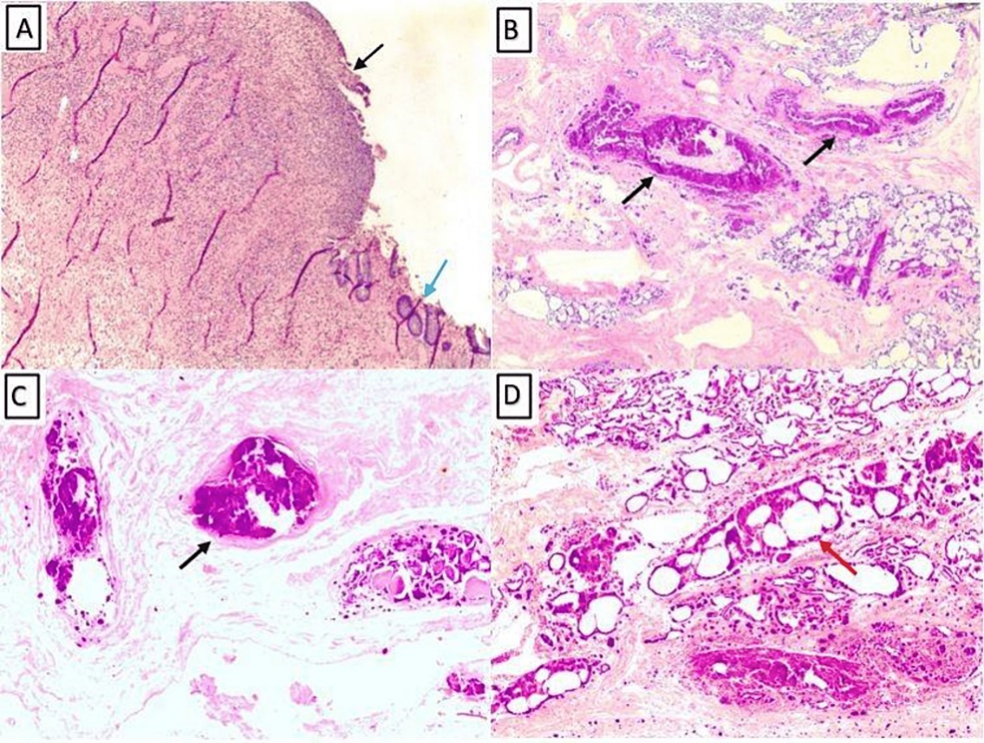
Histopathology findings on H&E stain of the resected colon A: Ulceration of the colon epithelium with complete loss of architecture indicating ischemic necrosis (black arrow) with adjacent ongoing ischemic damage with some remnant mucosal gland (blue arrow) (40× magnification). B and C: Extensive basophilic calcification of the blood vessels in the peri-colonic tissue with the absence of inflammatory cell infiltrates in the surrounding tissue (black arrows) (40× and 100× magnification, respectively). D: Fat necrosis and adjacent tissue calcification in the peri-colonic tissue (red arrow) (100× magnification). H&E: hematoxylin and eosin

The patient underwent exploratory laparotomy with partial colectomy of the descending and sigmoid colon due to intramural ischemia. Histopathology revealed architectural simplification and necrosis of the colonic mucosa and extensive calcification of the arterioles and pericolonic tissues without any signs of active inflammation consistent with calciphylaxis (Figure 3). The postoperative period was complicated with fluid collection inferior to the right lobe of the liver, which was drained. The patient also underwent sacral decubitus ulcer debridement with osteomyelitis changes in the coccyx. He received multiple broad-spectrum antibiotics throughout the course of treatment, which were tailored per culture sensitivity. He also underwent regular hemodialysis along with sodium thiosulphate infusion for calciphylaxis. The patient improved clinically without any further complications. A goal of care discussion was done, and the patient was discharged home under hospice care per his request.

## Discussion

The incidence of calciphylaxis per a study done in 2016 was estimated to be 3.5 new cases per 1,000 patient-years among chronic hemodialysis patients [12]. The most common cause of mortality is sepsis secondary to wound infection [7]. Various suggested risk factors include hyperparathyroidism, hyperphosphatemia, female gender, obesity, diabetes, autoimmune conditions, warfarin use, calcium-based binders, and vitamin D analog use [13]. Our patient had the risk factors of hyperparathyroidism and diabetes. The common sites involved include sites with increased adipose tissues such as the upper and lower extremities and the trunk [14]. Penile involvement is reported in approximately 6% of patients [15]. Although the pathogenesis is not clear, it is hypothesized to occur in two stages. The first is due to microvessel calcification leading to chronic ischemia, and the second stage is an endothelial injury resulting in infarction. Microvessel calcification occurs due to an imbalance between promoters (e.g., bone morphogenetic protein 2 and 4) and inhibitors (e.g., carboxylated matrix Gla protein, inorganic pyrophosphate, and fetuin-A) of calcification [1].

Although no specific laboratory findings are diagnostic, increased parathyroid hormone (PTH), calcium, and phosphorus may be seen. The gold standard of diagnosis is usually by skin biopsy revealing calcification of the medial layer of arteries and arterioles [16]. However, a skin biopsy is spared for the risk of inducing a new nonhealing wound resulting in infection and is reserved for atypical presentation [17]. Radiological findings may support this, but the accuracy of diagnosis has not been evaluated yet. Differential diagnosis includes atherosclerosis, warfarin-induced necrosis, vasculitis, cholesterol embolization, and cellulitis [18]. The differentiating feature between vasculitis and atherosclerosis is that CUA has intact peripheral pulses, bilateral necrosis, and more frequent involvement of the upper extremity [19].

Our patient had a confirmed diagnosis of systemic calciphylaxis complicated by penile amputation and was being treated with sodium thiosulphate. His physical examination was classic for uncontrolled calciphylaxis, and abdominal CT revealed extensive calcification of the intra-abdominal arteries, the degree of ischemia involving the sigmoid up to the mid-descending colon. Histopathology of the resected colon also showed signs of calcification without any signs of inflammatory infiltrates, ruling out vasculitis. Thus, we highly suggest calcific uremic arteriopathy as a cause of colon ischemia and necrosis resulting in perforation and abdominal infection.

There are no definitive treatment guidelines for CUA given the low prevalence and high mortality, making it difficult to perform large clinical trials. The treatment is the same for all manifestations of calciphylaxis, irrespective of the site involved. It is usually multidisciplinary, involving nephrology, surgery, infectious disease, wound care, and finally pain and palliative care specialists [13]. The main components involve pain control, wound care, resection of the affected area if needed, mitigation of risk factors including correction of calcium and phosphorus, and hyperparathyroidism, dialysis optimization, and medications such as sodium thiosulfate, bisphosphonates, and calcimimetics.

A systematic search of the literature for available case reports related to gastrointestinal manifestations yielded nine case reports, the summary of which is listed in Table 1 [3-11]. Presentation varied between GI bleeding and acute abdomen with pneumoperitoneum, and the most common diagnostic studies utilized were CT of the abdomen or gastrointestinal scope. Two case reports stated extensive visceral calciphylaxis causing splenic and cerebral infarct. The duration of hemodialysis varied from one month to three years. Histopathology findings of the gastrointestinal mucosa included ischemic necrosis with extensive blood vessel calcification. Treatment varied from optimization of hemodialysis, sodium thiosulphate, resection of necrotic tissues, and supportive management.

**Table 1:** Summary of case reports on gastrointestinal calciphylaxis BMI: body mass index, DM: diabetes mellitus, EGD: esophagogastroduodenoscopy, GI: gastrointestinal, HD: hemodialysis, HP: histopathology, Na thiosulphate: sodium thiosulphate, PTH: parathyroid hormone, PD: peritoneal dialysis, PPI: proton pump inhibitor

Author name	Tamura et al., 1995 [3]	Rivera-Nieves et al., 2002 [4]	Gupta et al., 2015 [5]	Mulgund et al., 2016 [6]	Machavarapu et al., 2018 [7]	Kawaji et al., 2018 [8]	Kang et al., 2019 [9]	Tan et al., 2020 [10]	Cochrane et al., 2021 [11]
Patient age	50	63	66	58	57	36	63	40s	57
Gender	Female	Female	Female	Male	Female	Female	Female	Male	Female
BMI	-	-	-	-	-	-	-	26	-
DM	Yes	Yes	Yes	-	Yes	Yes	Yes	Yes	-
Warfarin use	-	-	-	-	-	-	Yes	-	-
Dialysis duration	1 month	4 months HD + unknow PD	Unknown	Unknown	17 months PD	Unknown	3 years	15 months	Unknown
PTH	Low	Normal	High	-	-	Normal	High	High	-
Calcium	Normal	Normal	Normal	Normal	Normal	-	Normal	Normal	-
Phosphorus	High	High	High	Normal	Normal	High	Normal	High	-
GI manifestation	Melena, diffuse ulcer in the stomach and sigmoid colon, ischemic colitis	Acute abdomen with perforation and ischemic colitis	Lower GI bleed and pneumoperitoneum	Abdominal pain with gastric pneumatosis	Upper GI bleed	Acute abdomen with extensive small bowel pneumatosis	Bleeding, rectal necrosis, gastric ulcer	Cecal perforation, mesenteric ischemia	Gastric ulcer bleeding
Other visceral involvement	Splenic infarct, cerebral infarct	-	-	-	-	-	Splenic infarct	-	-
Diagnostic testing	Gastrointestinal fibroscopy, X-ray: pipe stemming shape of mesenteric arteries	Radionucleotide bone: scan uptake in soft tissue	CT of the abdomen severe circumferential calcification of abdominal arteries and pneumoperitoneum	EGD diffuse hemorrhagic and necrotic gastric ulcer	EGD esophageal ulcer	CT of the abdomen small bowel pneumatosis	CT of the abdomen abdominal vessel calcification, radionucleotide bone scan uptake in soft tissue	None	EGD pre-pyloric ulcer
HP finding	Diffuse medial calcification of the gastric and mesenteric arteries	Mural calcification of medium to large blood vessels in the mesentery	None	Acute ulcerative gastritis and intravascular calcium phosphate crystals	Acute ulceration with concentric calcification of capillary walls	Transmural ischemic necrosis with ulceration	Rectal biopsy necrotic tissue with patchy calcification	Mesenteric tissue arterial narrowing, intimal/medial hyperplasia,	Calcium deposition within lamina propria capillaries
Treatment given	Vitamin D3, prednisone	Surgical resection of transverse colon	Hyperbaric oxygen, lidocaine, and Na thiosulfate intradermal	PPI and carafate	Na thiosulphate, PPI	Resection	Na thiosulphate, parathyroidectomy	Na thiosulphate, vitamin K, surgical resection	PPI
Mortality	Dead	Dead	Dead	Alive	Dead	Dead	Alive	Alive	Alive

Although the prognosis is poor, our patient responded well to resection and debridement for osteomyelitis of the coccyx. Plastic surgery evaluation was also considered, but given the patient’s decision, he was discharged under hospice care.

## Conclusions

Calciphylaxis is a lethal disease despite treatment. Visceral, penile, and proximal extremity involvement has been associated with more significant morbidity and mortality. Although gastrointestinal calciphylaxis is favored in patients with ischemic colitis or GI hemorrhage in the background of calciphylaxis and histopathology finding of extensive arterial calcification, differential diagnosis including atherosclerosis, warfarin-induced necrosis, vasculitis, and cholesterol embolization should be considered.
